# Supplementary material for: Anti-inflammatory and pro-proliferative effects of fasudil in human trisomy 21 neural progenitor cells
Source: bioRxiv. 2026 Mar 20:2026.03.19.712922. Preprint. [Version 1] doi: 10.64898/2026.03.19.712922 (PMC13015371; doi:10.64898/2026.03.19.712922)
Supplement: 1 [file NIHPP2026.03.19.712922V1-supplement-1.pdf]

bioRxiv preprint doi: https://doi.org/10.64898/2026.03.19.712922; this version posted March 20, 2026. The copyright holder for this preprint (which was not certified by peer review) is the author/funder. This article is a US Government work. It is not subject to copyright under 17 USC 105 and is also made available for use under a CC0 license.

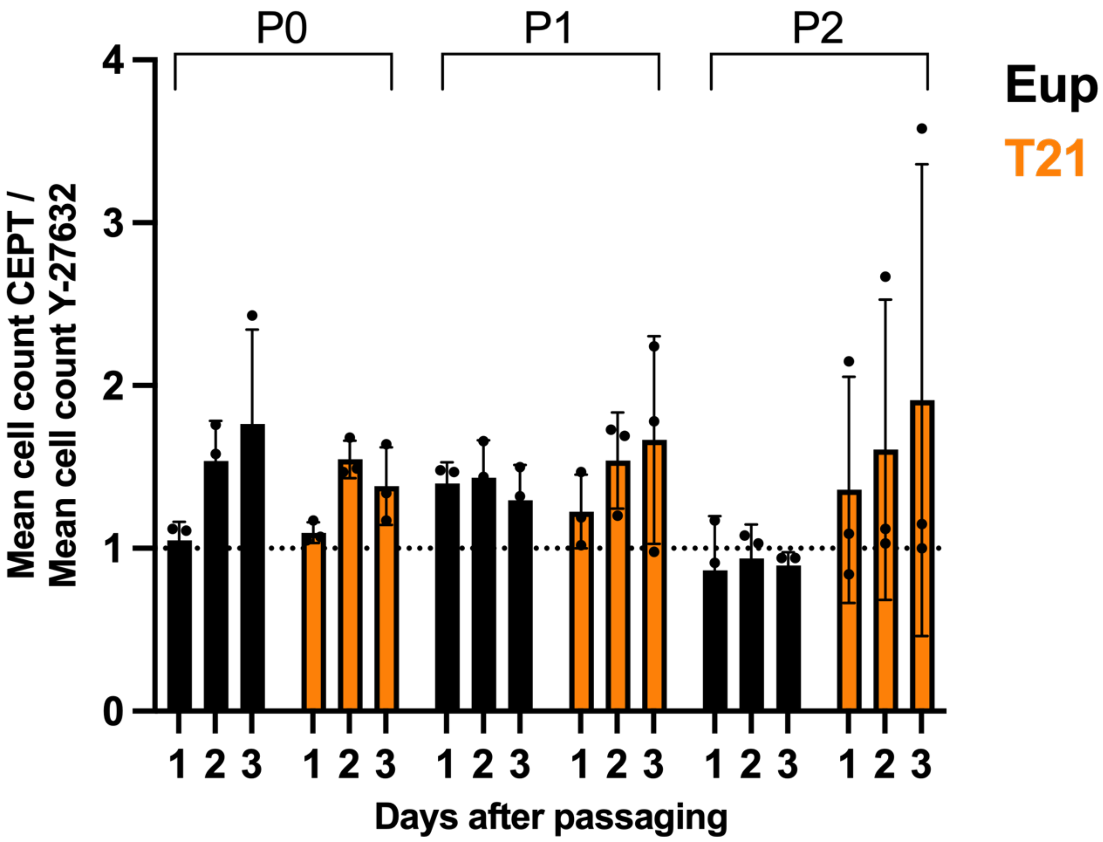

Supplemental figure S1. **Passaging with CEPT improved growth during neural induction of NPCs from iPSCs.** More cells survived passaging with CEPT supplementation in comparison to Y-27632 supplementation in Eup and T21 lines. Ratios of CEPT-treated cell count to Y-27632 cell count in the same line are shown at one, two, and three days after passages 0, 1, and 2 (P0, P1, and P2, respectively). Ratio values >1 indicate greater cell numbers in CEPT-treated relative to Y-27632-treated lines. N = 3 Eup lines (EU6, EU7, EU12) and 3 T21 lines (TS3, TS4, TS8). Each data point = the mean of 3 replicates. Black = Eup lines, orange = T21 lines.

bioRxiv preprint doi: https://doi.org/10.64898/2026.03.19.712922; this version posted March 20, 2026. The copyright holder for this preprint (which was not certified by peer review) is the author/funder. This article is a US Government work. It is not subject to copyright under 17 USC 105 and is also made available for use under a CC0 license.

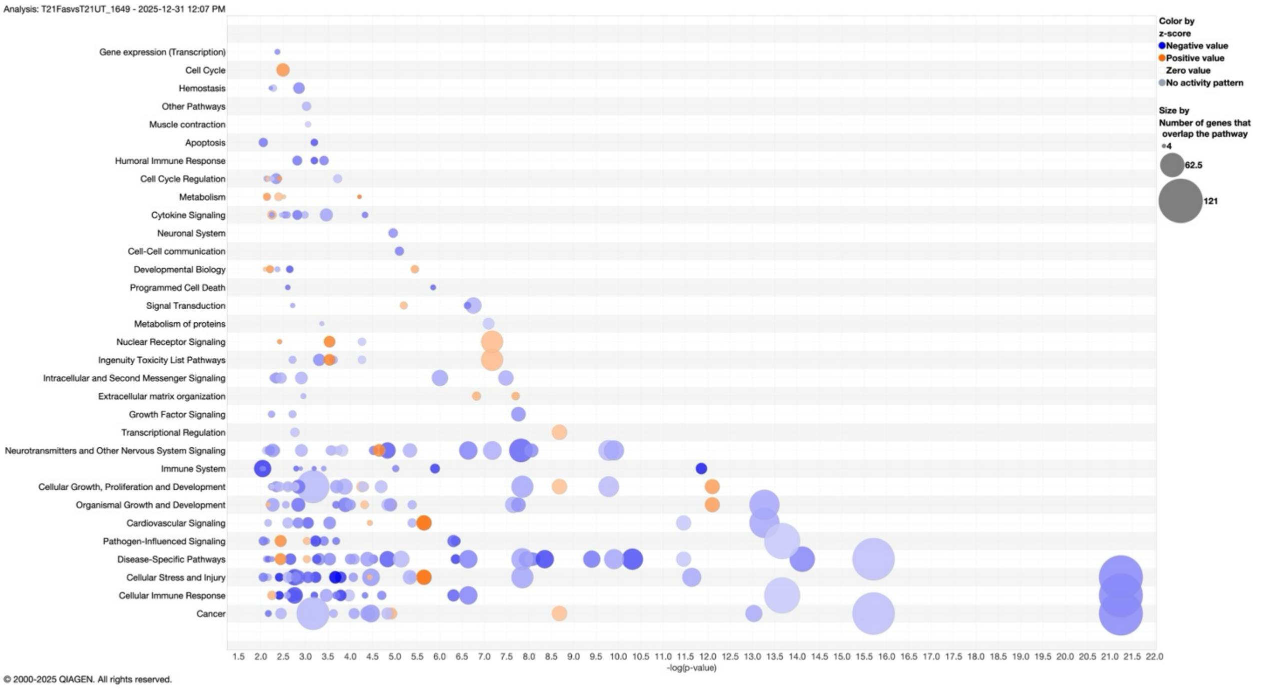

Supplemental figure S2. Significantly enriched pathways in fasudil-treated T21 NPCs. Ingenuity Pathway Analysis for the 1649 T21 DEGs showed categories of significantly enriched pathways, including downregulation of numerous cellular immune response / immune system and cellular stress and injury pathways. Blue = negative Z score / pathway downregulation, and orange = positive Z score / pathway upregulation. Circle size represents the number of DEGs in the pathway.
